# Population structure and adaptive variation of *Helichrysum italicum* (Roth) G. Don along eastern Adriatic temperature and precipitation gradient

**DOI:** 10.1038/s41598-021-03548-6

**Published:** 2021-12-21

**Authors:** Tonka Ninčević, Marija Jug-Dujaković, Martina Grdiša, Zlatko Liber, Filip Varga, Dejan Pljevljakušić, Zlatko Šatović

**Affiliations:** 1 Department for Plant Sciences, Institute for Adriatic Crops and Karst Reclamation, Put Duilova 11, 21000 Split, Croatia; 2 Department of Seed Science and Technology, Faculty of Agriculture, University of Zagreb, Svetošimunska c. 25, 10000 Zagreb, Croatia; 3 Centre of Excellence for Biodiversity and Molecular Plant Breeding (CoE CroP-BioDiv), Svetošimunska c. 25, 10000 Zagreb, Croatia; 4 Department of Biology, Faculty of Science, University of Zagreb, Marulićev trg 9a, 10000 Zagreb, Croatia; 5 Institute for Medicinal Plants Research, “Dr. Josif Pančić”, Tadeuša Koščuška 1, 11000 Belgrade, Serbia

**Keywords:** Plant sciences, Genetics, Plant genetics, Population genetics

## Abstract

Immortelle (*Helichrysum italicum* (Roth) G. Don; Asteraceae) is a perennial plant species native to the Mediterranean region, known for many properties with wide application mainly in perfume and cosmetic industry. A total of 18 wild *H. italicum* populations systematically sampled along the eastern Adriatic environmental gradient were studied using AFLP markers to determine genetic diversity and structure and to identify loci potentially responsible for adaptive divergence. Results showed higher levels of intrapopulation diversity than interpopulation diversity. Genetic differentiation among populations was significant but low, indicating extensive gene flow between populations. Bayesian analysis of population structure revealed the existence of two genetic clusters. Combining the results of *F*_*ST*_ - outlier analysis (Mcheza and BayeScan) and genome-environment association analysis (Samβada, LFMM) four AFLP loci strongly associated with the bioclimatic variables Bio03 Isothermality, Bio08 Mean temperature of the wettest quarter, Bio15 Precipitation seasonality, and Bio17 Precipitation of driest quarter were found to be the main variables driving potential adaptive genetic variation in *H. italicum* along the eastern Adriatic environmental gradient. Redundancy analysis revealed that the partitioning of genetic variation was mainly associated with the adaptation to temperature oscillations. The results of the research may contribute to a clearer understanding of the importance of local adaptations for the genetic differentiation of Mediterranean plants and allow the planning of appropriate conservation strategies. However, considering that the identified outlier loci may be linked to genes under selection rather than being the target of natural selection, future studies must aim at their additional analysis.

## Introduction

The Mediterranean basin area is one of the largest biodiversity hotspots in the world^1^. It occupies only 2% of the world's land area and is the habitat for great number of species^2^. Heterogeneous environments and numerous past events are responsible for significant plant diversity and a large number of endemic species in this area^2^. The Mediterranean basin is the meeting point of three continents; a place where humans began to exert their influence on biota and environment very early^3^. Today, human activity continues to pose a threat to the biodiversity in the Mediterranean, mainly because population density is highest along the coastal region, where numerous refugia have been discovered^4^. Refugia are known to act as climate-stable zones that are crucial for the long-term survival of species and genetic diversity. Populations that survived in glacial refugia show high rarity index values (DW; frequency down-weighted marker values) and high genetic diversity due to long-term isolation^5^. Populations that emerged after the Last Glacial Maximum show lower genetic variation^6^ and lower rarity as a result of consecutive founder events during postglacial colonization^7,8^. It is very important to preserve these areas of numerus refugia, which are vital for the evolutionary processes of Mediterranean plant species^4^.

Climate change, overexploitation of natural populations, degradation, and fragmentation of natural habitats are the direct drivers affecting biodiversity^9,10^, and the impact of each factor on the evolutionary processes of a species must be considered for appropriate conservation planning.

Local adaptation is an important tool of species through which natural populations are phenotypically and genotypically separated to better adapt to new habitat conditions and survive^11,12^. From a theoretical perspective, the interaction between gene flow and natural selection has been well studied^13^, but there are still too few empirical studies to clarify the establishment of local adaptation and maintenance at the molecular level in natural populations. For non-model perennial and outcrossing species, genomic and evolutionary research is more difficult to conduct, and the genetic background of adaptive traits remains poorly understood^14,15^. Plant species that have wide geographic distribution and thereby are exposed to a range of environmental conditions, are often locally adapted, in part because of higher gene flow^14–16^. Discovering the mechanisms of adaptability of plant species is important for predicting their survival due to climate change or to what extent certain plant species will develop or activate adaptive mechanisms to the new conditions^17,18^.

Genetic signatures of natural selection are revealed by genome scanning^19–21^. Genotyping of random loci throughout the genome allows the detection of outlier loci characterized by a higher degree of differentiation between populations than would be expected for neutral loci^20,22–25^. Identification of genetic regions under selection is commonly performed using two methods: outlier loci detection and genome-environment association analyzes. Both approaches assume that only a small number of loci are under selection^26^. Detection of outlier loci is a population-level analysis based on estimates of population genetic differentiation (*F*_*ST*_), whereas genome-environment analysis examines correlations between population allele frequencies and environmental variables^27–29^. Using these methods, local adaptation along environmental gradients was studied in several plant species: *Eruca sativa* Mill.^30^; *Eucalyptus camaldulensis* Dehnh.^31^; *Abies alba* Mill.^32^; *Liriodendron chinense* (Hemsl.) Sarg.^21^; *Geropogon hybridus* (L.) Sch.Bip.^33^; *Diplotaxis harra* (Forssk.) Boiss.^34^, *Populus tremula* L.^35^, and *Festuca pallescens* (St.-Yves) Parodi^36^.

To study local adaptation along the eastern Adriatic environmental gradient characterized by increasing temperatures and precipitation, we chose *H. italicum*, a typical Mediterranean representative of thermophilic species. The species is naturally distributed along the eastern Adriatic coast and on the islands along the northwest-southeast environmental gradient. It grows on calcareous and well-drained soils: from sea level to 2200 m a.s.l.^37^. It is an outcrossing, thermophilic perennial and entomophilous plant species. As an anemochorous species, it is easily dispersed by wind, which contributes significantly to the spread of the species. It belongs to the genus *Helichrysum* Mill. and the family Asteraceae. The genus *Helichrysum* has more than 500 species worldwide^38^, with the species *H. italicum* distributed in the Mediterranean region. Herrando-Moraira et al.^39^ revised the classification of the entire *H. italicum* complex and proposed a new division of the *H. italicum* subspecies: (1) *H. italicum* subsp. *italicum* (grows in Italy, Croatia, on the eastern Mediterranean coast of France and Corsica, in Bosnia and Herzegovina, Greece (Aegean islands and Cyprus), (2) *H. italicum* ssp. *microphyllum* (endemic to Crete), (3) *H. italicum* ssp. *siculum* (endemic to Sicily), and (4) *H. italicum* ssp. *tyrrhenicum* (disjunct distribution area between islands Corsica, Sardinia, Mallorca, and Dragonera islet). Numerous studies on the medicinal properties and biological activity of *H. italicum* were conducted, and are summarized in Guinoiseau et al.^40^, Maksimovic et al.^41^, and Ninčević et al.^42^. There have been several studies on the genetic diversity of *Helichrysum* spp.^37,43–46^ but none of them focused on the eastern Adriatic natural populations. Owing to many beneficial properties it is used in many commercial products, which increased the demand for *H. italicum*. This has led to overexploitation of natural populations, especially on the eastern Adriatic coast, where the collection of *H. italicum* contributes significantly to the local economy (e.g. in Croatia immortelle is the most collected of all other wild medicinal species). In general, the use of medicinal and aromatic plants (MAP) has a long tradition on the eastern Adriatic coast. Commercial gathering is an important source of main or additional income in many regions^47,48^. Wild collection is still the main way to supply the market with medicinal and aromatic plants, as production is still insufficient. The negative impacts of wild collection are manifested in the loss of biodiversity, inconsistent plant material and lower prices. Increasing demand and resulting overexploitation, destructive harvesting techniques along with habitat loss and degradation^49^ put great pressure on many medicinal plants, leading to a decrease in genetic diversity and a reduction in the species potential to respond to environmental changes^47,50^. Therefore, it is of great importance to assess genetic biodiversity for efficient conservation of plant genetic resources and their use in plant breeding programs^48^.

In this research, an AFLP genome scan was performed on a total of 18 natural *H. italicum* populations systematically sampled along the eastern Adriatic environmental gradient to elucidate genetic diversity within and among populations, identify candidate loci subjected to selection, and investigate their relationship with environmental conditions. Possible effects of past climatic variability and overexploitation on the genetic diversity and structure of natural *H. italicum* populations are considered, as well as the importance of local adaptations to climatic conditions as important promoters of genetic differentiation.

## Results

### Within‑population diversity

Diversity within and between 18 populations of *H. italicum* was analyzed using AFLP markers. Four combinations of primers resulted in 693 polymorphic AFLP markers. The overall error rate for all combinations of selective PCR primers was 4.87%.

**Figure 1:**
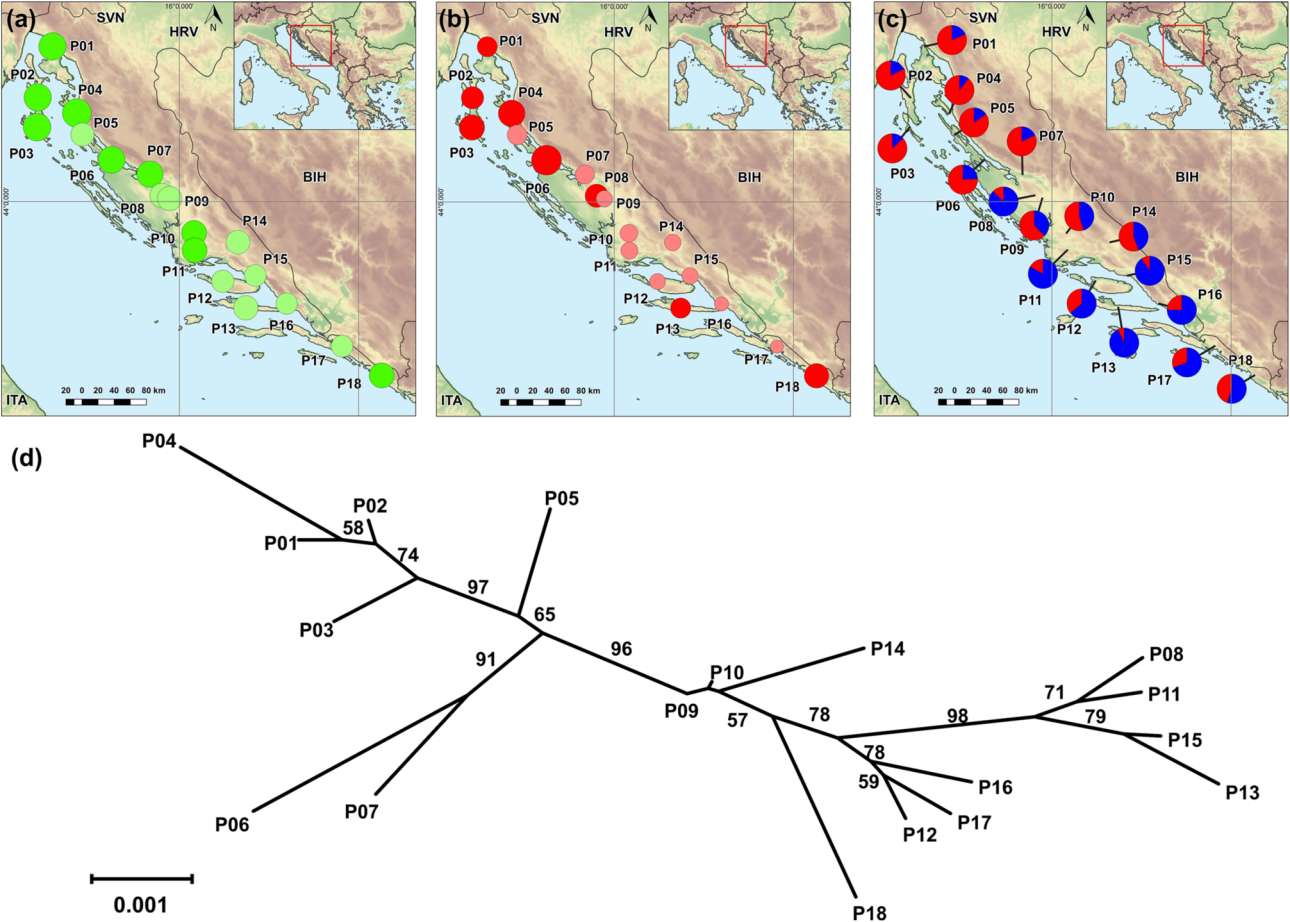
Genetic diversity and relationships among 18 *H. italicum* populations along eastern Adriatic coast: (**a**) Shannon’s information index, (**b**) Frequency down-weighted marker values, (**c**) Genetic structure derived from Bayesian analysis using STRUCTURE at *K* = 2, (**d**) the Fitch-Margoliash tree based on Nei’s genetic distance matrix between populations. Bootstrap values greater than 50% based on 1000 pseudorepeats are marked on the branches. In (**a**) and (**b**), the size of the dots is directly proportional to the depicted values. Maps were generated using QGIS 3.10.7 (https://qgis.org/).

The Shannon information index ranged from 0.330 (P16 Živogošće) to 0.383 (P04 Rab) with an average value of 0.355 (Table 1; Fig. 1a). The average frequency of rare alleles (DW) was 50.82. The highest frequency of rare alleles (DW) was 76.10 for the population P06 Miškovići, Pag, and the lowest 33.53 for population P17 Slano (Fig. 1b). Expected heterozygosity (*H*_*E*_) was calculated within each of the analyzed populations based on gene frequencies assuming equilibrium according to Hardy–Weinberg (Table 1). The lowest gene diversity was found in the population P16 Živogošće (*H*_*E*_ = 0.134), and the highest in the population P04 Rab (*H*_*E*_ = 0.153). The total gene diversity (*H*_*T*_) was 0.147, and an average gene diversity within populations (*H*_*W*_) was 0.142.

**Table 1:** Sampling locations and genetic diversity revealed by AFLP markers in 18 *H. italicum* populations from eastern Adriatic coast.

No	Population	Latitude (N)^a^	Longitude (E)^a^	Elevation (m a.s.l.)	n	*P%*	*N*_*pr*_	*I*	*H*_*E*_	*DW*
P01	Krk	45.23	14.58	36	24	0.636	0	0.373	0.145	51.13
P02	Cres	44.83	14.42	248	25	0.649	1	0.373	0.148	56.79
P03	Lošinj	44.59	14.41	73	25	0.654	1	0.375	0.148	64.59
P04	Rab	44.70	14.86	44	25	0.633	1	0.383	0.153	67.94
P05	Pag (Zrće)	44.53	14.92	18	24	0.582	0	0.344	0.141	50.58
P06	Pag (Miškovići)	44.33	15.24	42	25	0.631	0	0.370	0.150	76.10
P07	Obrovac	44.22	15.67	137	24	0.615	0	0.366	0.148	48.73
P08	Benkovac	44.05	15.81	203	25	0.593	1	0.353	0.141	59.22
P09	Kistanje	44.02	15.89	302	25	0.608	0	0.352	0.138	41.88
P10	Unešić	43.75	16.16	390	24	0.610	0	0.356	0.140	45.05
P11	Seget	43.61	16.17	426	25	0.610	0	0.356	0.139	43.44
P12	Brač	43.36	16.48	290	25	0.571	0	0.337	0.137	40.85
P13	Hvar	43.14	16.74	361	25	0.600	0	0.353	0.141	51.56
P14	Sinj	43.67	16.65	343	25	0.589	0	0.349	0.140	42.63
P15	Omiš	43.40	16.85	116	25	0.569	1	0.334	0.136	42.31
P16	Živogošće	43.18	17.20	125	25	0.551	0	0.330	0.134	36.00
P17	Slano	42.83	17.82	338	23	0.548	0	0.332	0.136	33.53
P18	Cavtat	42.59	18.26	525	25	0.592	0	0.357	0.143	62.46

^a^N-North; E-East; Coordinates are in degree decimal format; *n* - sample size; *%P* - proportion of polymorphic bands; *Npr - *number of private bands; *I* - Shannon’s information index; *H*_*E*_ - gene diversity of population assuming Hardy-Weinberg equilibrium; *DW* - frequency down-weighted marker values.

### Population differentiation and structure

The Wright coefficient of genetic differentiation (*F*_*ST*_) at the level of all populations was 0.036. The highest value of *F*_*ST*_ was calculated between populations P04 Rab and P13 Hvar (*F*_*ST*_ = 0.069), and the lowest between populations P09 Kistanje and P10 Unešić (*F*_*ST*_ = 0.002).

The results of molecular variance analysis showed that 93.08% of diversity belongs to diversity within-population, and 6.92% to diversity between populations. The results were obtained after 10,000 permutations with significance *P*(*φ*) < 0.001. A matrix of *φ*_*ST*_ values was calculated between all pairs of populations by individual analyzes (Table S1). The *φ*_*ST*_ values ranged from 0.010 between P09 Kistanje and P10 Unešić to 0.127 between populations P05 Zrće, Pag, and P13 Hvar, with a mean of 0.068.

Assuming Hardy-Weinberg equilibrium, a Nei’s genetic distance was calculated between 18 *H. italicum* populations. The Nei’s distance varied from 0.0003 between populations P09 Kistanje and P10 Unešić to 0.0128 between populations P04 Rab and P13 Hvar, with a mean value of 0.0062. The Fitch-Margoliash tree grouped the populations according to their geographical origin. This grouping showed strong differentiation between the populations of Kvarner bay (P01 Krk, P02 Cres, P03 Lošinj, P04 Rab, P05 Pag (Zrće), P06 Pag (Miškovići), and P07 Obrovac) and the populations of Dalmatia (P08 Benkovac, P09 Kistanje, P10 Unešić, P11 Seget, P12 Brač, P13 Hvar, P14 Sinj, P15 Omiš, P16 Živogošće, P17 Slano, and P18 Cavtat) supported by bootstrap value of 96% (Fig. 1d).

The results of Bayesian model-based clustering using STRUCTURE are shown in Fig. 1c. The log-likelihood of the hypotheses [*ln P*(*X|K*)] and the rate of change between successive values of *K* (Δ*K*) for different numbers of ancestral populations (*K* = 1–11) were obtained. The highest value of *ΔK* was found at *K* = 2 (Δ*K* 386.84) followed by *K* = 3 (Δ*K* = 101.29). For *K* values greater than three, the average [*ln P*(*X*|*K*)] values decreased, while the standard deviations between the different runs for each *K* increased considerably and, therefore, the Δ*K* values were notably lower (from 6.50 to 0.08) (Fig. S1).

The proportions of membership of each individual in each of the gene pools were calculated for *K* = 2 based on the run with the highest [*ln P*(X|K)], and populations were assigned to a particular cluster (A and B). The results were congruent with those obtained using the distance-based method. At *K* = 2 populations mainly from North Adriatic and Kvarner Bay (P01 Krk, P02 Cres, P03 Lošinj, P04 Rab, P05 (Zrće) Pag, P06 (Miškovići) Pag, and P07 Obrovac) were assigned to the cluster A, while populations from central and south of investigated area (Dalmatia) (P08 Benkovac, P11 Seget, P13 Hvar and P15 Omiš) were assigned to the cluster B.

As maximum *ΔK* at *K* = 2 could be an artifact resulting from significantly low likelihoods for *K* = 1^51^, we used the BAPS program to verify data obtained from STRUCTURE. The results showed agreement with those from STRUCTURE for *K* = 2. The best partitions obtained log marginal likelihoods of −89,691.83 at *P* = 1 (without using geographic coordinates as informative priors) and −90,102.87 at *P* = 1 (with spatially informative priors). Both analyzes, with or without spatially informative priors classified the populations identically into two clusters as revealed by the STRUCTURE analysis (Fig. S2).

The correlation between the matrix of *F*_*ST*_ / (1−*F*_*ST*_) values and the matrix of natural logarithms of geographic distances (in km) between the analyzed populations was *r* = 0.510 and was highly significant (*P*_Mantel_ < 0.0001). The coefficient of determination was *R*^2^ = 0.260, revealing that 26% of the genetic differentiation between the analyzed populations can be explained by their spatial distance (Fig. S3).

### Bioclimatic variation

**Figure 2:**
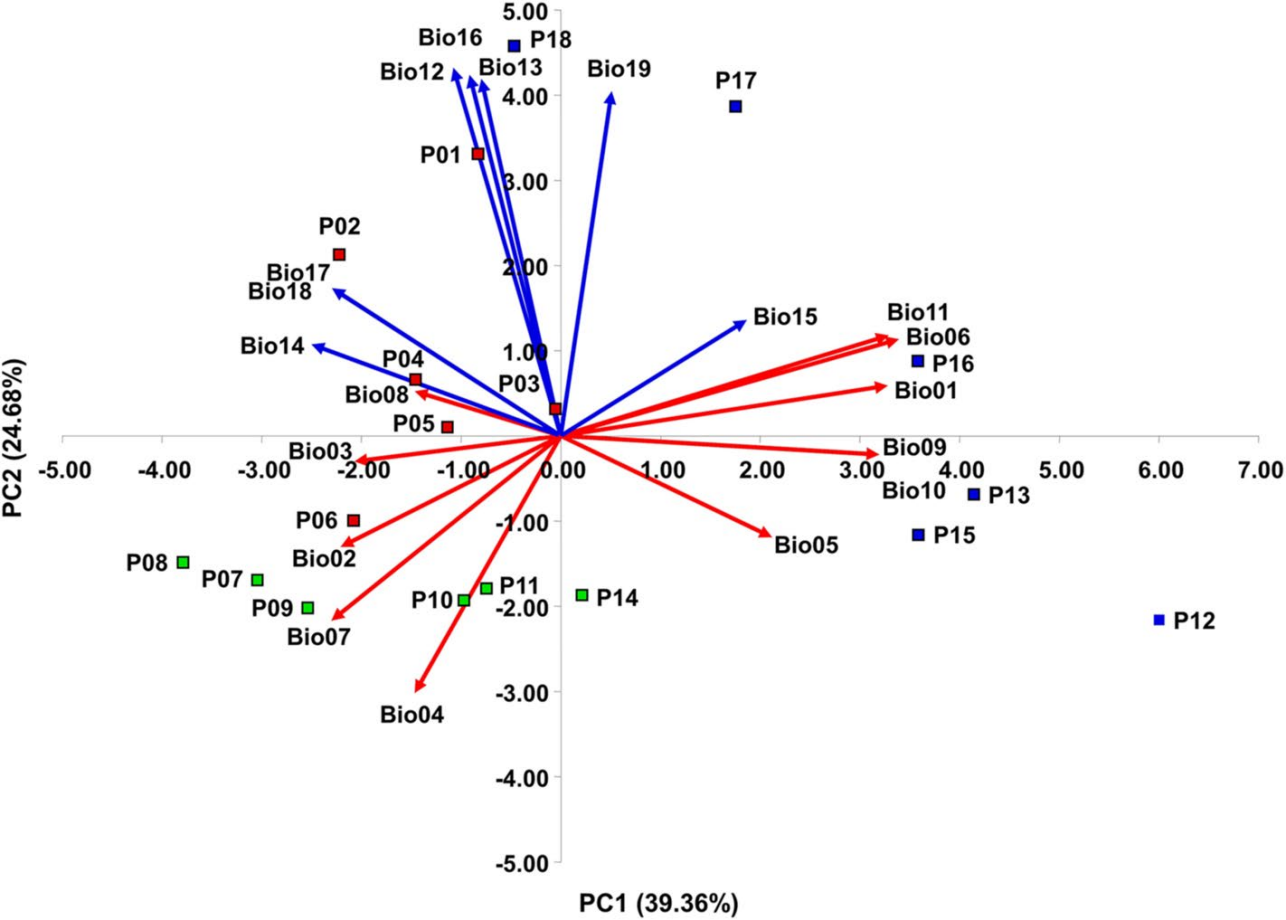
Biplot obtained by Principal Component Analysis (PCA) based on 19 bioclimatic variables for 18 *H. italicum* sampling sites. Red vectors represent temperature-related variables and blue vectors precipitation-related variables. Northern Adriatic *H. italicum* populations (P01–P06) are colored in red, central populations in green (P07–P10) and southern populations (P11–P18) in blue color.

Nineteen bioclimatic variables accounted for in this study were highly correlated. A strong positive correlation (*r* > 0.70) was found in 26 cases, and in five cases a strong negative correlation (*r* < −0.70), out of 171 pairs examined, respectively (Table S2. Principal component analysis (PCA), based on the correlation matrix, showed that the first four principal components had eigenvalues greater than 1 and together explained 93.82% of the variance (Table 2). The first principal component explained 39.36% of the total variance. A strong positive correlation with the first principal component (PC1) was found for five environmental variables (Bio01 Average annual temperature, Bio06 Minimum temperature of the coldest month, Bio09 Average temperature of the driest quarter, Bio10 Average temperature of the warmest quarter, Bio11 Average temperature of the coldest quarter). The second principal component explained 24.48% of the total variance and was positively correlated with four environmental variables (Bio12 Annual precipitation, Bio13 Precipitation in the wettest month, Bio16 Precipitation in the wettest quarter, and Bio19 Precipitation in the coldest quarter). The first principal component separated the southern Adriatic populations (P16 Živogošće, P15 Omiš, P13 Hvar and P12 Brač) from sampled sites characterized by higher temperatures and lower precipitation, from the northern Adriatic populations (P01 Krk, P02 Cres, P03 Lošinj, P04 Rab, P05 Zrće, Pag and P06 Miškovići, Pag) and central Adriatic populations (P14 Sinj, P11 Seget, P10 Unešić, P09 Kistanje, P08 Benkovac, and P07 Obrovac) where lower temperatures and higher precipitation were recorded. The second principal component separated the populations from the Kvarner Bay where higher precipitation and lower temperatures were recorded, from the populations from central Dalmatia where higher temperatures and lower precipitation were recorded (Fig. 2).

**Table 2:** Correlations between 19 environmental variables (Bio1–Bio19) and the first four principal components.

No	Environmental variable	PC1	PC2	PC3	PC4
Bio01	Annual Mean Temperature	0,897***	0.127^ns^	0.268^ns^	0.322^ns^
Bio02	Mean Diurnal Range	−0.604**	−0.283^ns^	−0.361^ns^	0.624**
Bio03	Isothermality	−0.566*	−0.065^ns^	−0.380^ns^	0.599**
Bio04	Temperature Seasonality	−0.400^ns^	−0.654**	−0.028^ns^	0.180^ns^
Bio05	Max Temperature of Warmest Month	0.581*	−0.258^ns^	0.072^ns^	0.764***
Bio06	Min Temperature of Coldest Month	0.928***	0.246^ns^	0.273^ns^	0.011^ns^
Bio07	Temperature Annual Range	−0.629**	−0.470*	−0.258^ns^	0.550*
Bio08	Mean Temperature of Wettest Quarter	−0.401^ns^	0.113^ns^	0.687**	0.034^ns^
Bio09	Mean Temperature of Driest Quarter	0.875***	−0.048^ns^	0.292^ns^	0.365^ns^
Bio10	Mean Temperature of Warmest Quarter	0.875***	−0.048^ns^	0.292^ns^	0.365^ns^
Bio11	Mean Temperature of Coldest Quarter	0.900***	0.255^ns^	0.237^ns^	0.211^ns^
Bio12	Annual Precipitation	−0.295^ns^	0.936***	−0.017^ns^	0.173^ns^
Bio13	Precipitation of Wettest Month	−0.217^ns^	0.907***	−0.306^ns^	0.151^ns^
Bio14	Precipitation of Driest Month	−0.684**	0.233^ns^	0.657**	0.142^ns^
Bio15	Precipitation Seasonality	0.511*	0.295^ns^	−0.789***	0.036^ns^
Bio16	Precipitation of Wettest Quarter	−0.250^ns^	0.918***	−0.216^ns^	0.200^ns^
Bio17	Precipitation of Driest Quarter	−0.627**	0.376^ns^	0.643**	0.177^ns^
Bio18	Precipitation of Warmest Quarter	−0.627**	0.376^ns^	0.643**	0.177^ns^
Bio19	Precipitation of Coldest Quarter	0.141^ns^	0.876***	−0.423^ns^	0.074^ns^
	Eigenvalue	7.48	4.69	3.39	2.27
	% of total variance	39.36	24.68	17.85	11.93
	Cumulative % of variance	39.36	64.04	81.89	93.82

*Ns* non-significant.

*Significant at *P* < 0.05.

**Significant at *P* < 0.01.

***Significant at *P* < 0.001.

### Adaptive genetic variation

**Figure 3:**
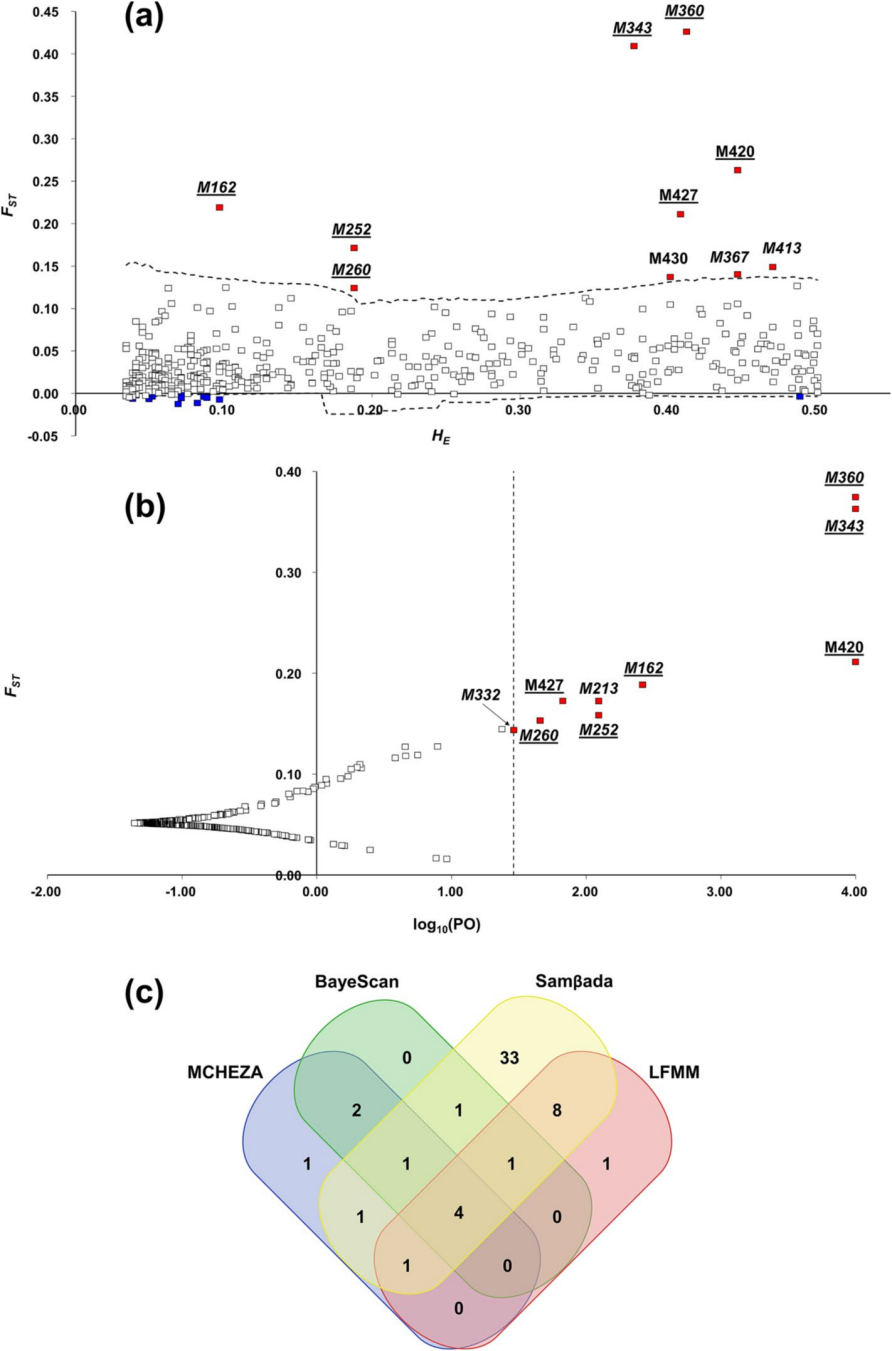
Identification of *F*_*ST*_ outlier loci using (**a**) Mcheza and (**b**) BayeScan and (**c**) the Venn diagram summarizing the number of loci identified as *F*_*ST*_ outlier loci by Mcheza and BayeScan and significantly associated with environmental variables by Samβada and LFMM. In (**a**) *F*_*ST*_ values were plotted against its heterozygosity (*H*_*E*_). The dashed lines represent the 99% confidence intervals. Loci under positive selection are indicated as red dots, those under balancing selection as blue dots, and neutral as grey dots. Loci under positive selection detected also by BayeScan are underlined while those identified by Samβada are shown in italics. In (**b**) *F*_*ST*_ values were plotted against the log_10_ of the posterior odds (PO). The vertical line shows the critical PO used for identifying outlier markers [FDR < 0.01; PO = 29,03; log_10_(PO) = 1463]. Loci under positive selection detected also by Mcheza are underlined while those identified by Samβada are shown in italics.

The outlier loci were detected on a set of 446 AFLP markers (markers with a frequency under 3% or above 97% were removed). With a 99% confidence level using the Mcheza program, a total of 21 outlier loci (4.71%) under possible selection were detected, of which ten (2.24%) were under positive selection (directional selection) and 11 (2.47%) under balancing selection (Fig. 3a). BayeScan identified nine (2.02%) loci under positive selection that exceeded the critical value of log_10_(PO) threshold used to determine the significance of loci that showed atypical values at a false-positive probability level of 0.01 [FDR < 0, 01; PO = 29.03; log_10_(PO) = 1.463]. No locus was under balancing selection (Fig. 3b). Mcheza and BayeScan together detected a set of seven (1.57%) markers that were potentially under positive selection. By calculating logistic regressions between all possible marker pairs and bioclimatic variable (8,474 models), the Samβada program revealed 184 (2.17%) significant models that included 50 (11.21%) markers associated with one to 13 bioclimatic variables. The Mcheza and BayeScan, identified 12 loci, and the Samβada program 41. Bioclimatic variable associated with more than 20 markers were Bio08 average temperature of the wettest quarter, Bio14 amount of precipitation in the driest month, Bio15 coefficient of precipitation variation, Bio17 amount of precipitation in the driest quarter, and Bio18 amount of precipitation in the warmest quarter (Table 3). Of 50 markers detected by Samβada, five markers were also identified by Mcheza and BayeScan to be under positive selection. Latent factor mixed model (LFMM) identified 50 markers associated with one to 15 bioclimatic variables. Out of the total 446 markers, four markers (0.89%) were identified by all four methods, as shown in the Wenn diagram (Fig. 3c). The bioclimatic variables Bio03 Isothermality, Bio08 Mean temperature of wettest quarter, Bio15 Precipitation seasonality, and Bio17 Precipitation of driest quarter, were correlated with the highest number of loci and may have played a key role in adaptive divergence in populations *H. italicum* in the eastern Adriatic (Table 3).

**Table 3:**
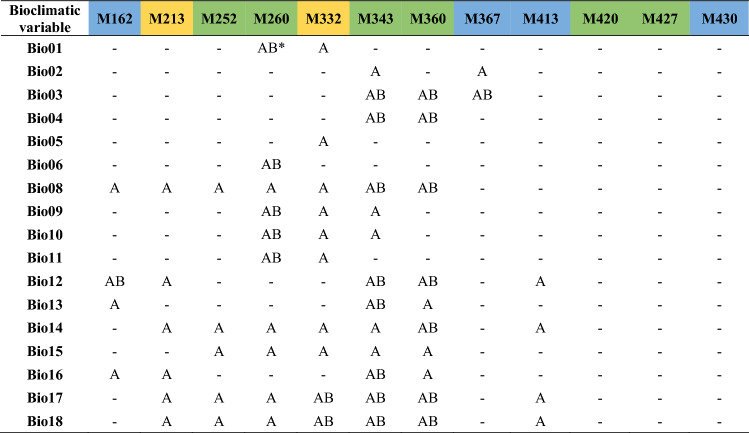
Potential *F*_*ST*_ outlier markers identified by both Mcheza and BayeScan (in green), exclusively by Mcheza (in blue), and exclusively by BayeScan (in yellow), along with their Association with bioclimatic variables detected using Samβada and LFMM.

*A represents significant correlation of outlier loci (either identified by Mcheza or Bayescan) and bioclimatic variables as revealed by Samβada; **B** represents significant correlation of outlier loci (either identified by Mcheza or Bayescan) and bioclimatic variables as revealed by LFMM.

**Figure 4:**
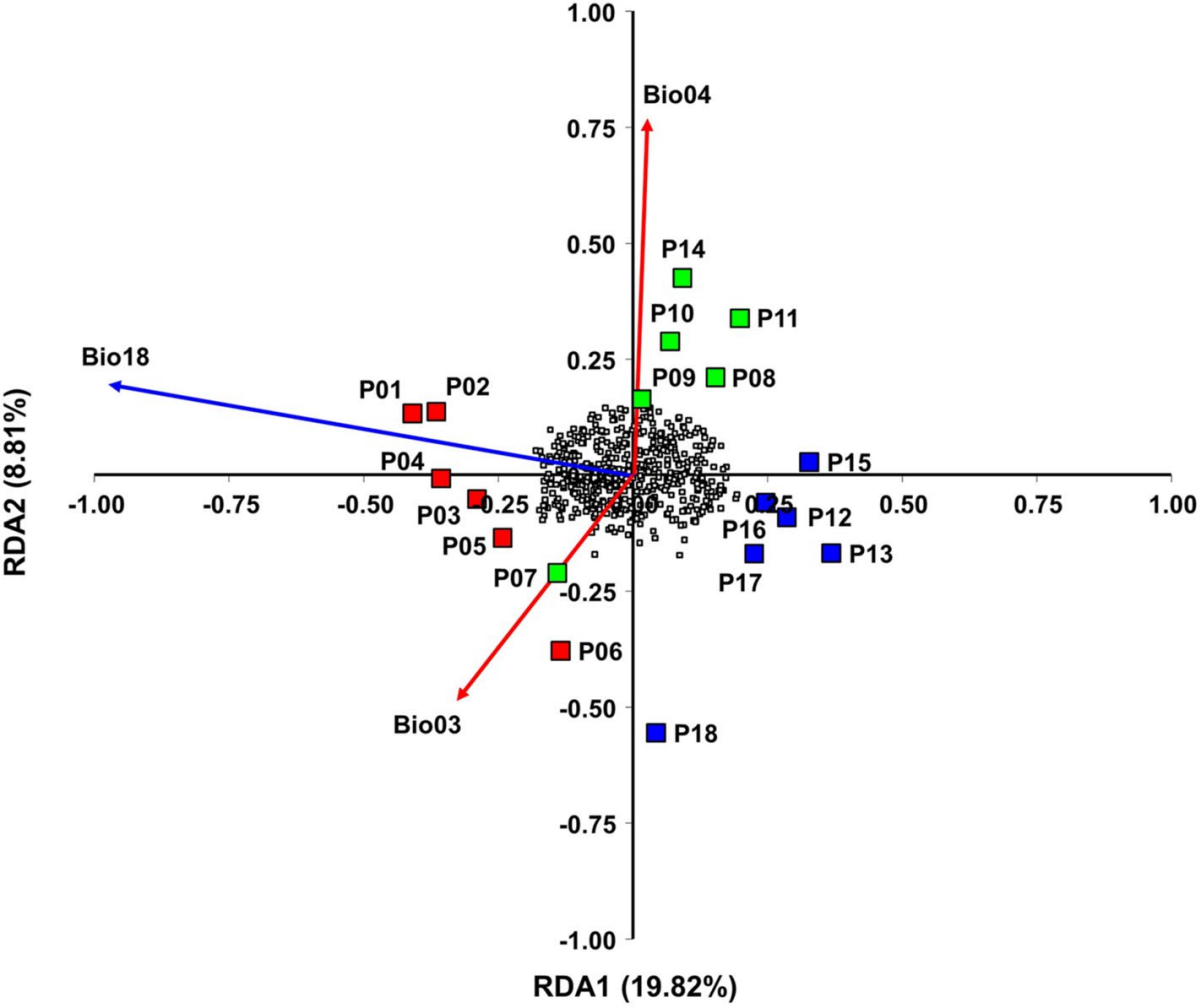
Triplot obtained by Redundancy analysis (RDA) based on three bioclimatic variables included in the optimal RDA model showing the relative contribution of bioclimatic variables in shaping the genetic structure of 18 *H. italicum* populations. Red vectors represent temperature-related variables (Bio3 and Bio04) and blue vector precipitation-related variable (Bio18). Northern Adriatic *H. italicum* populations (P01–P06) are colored in red, central populations in green (P07–P10) and southern populations (P11–P18) in blue color. Small empty boxes represent AFLPs.

Six uncorrelated bioclimatic variables, four temperature-related (i.e., Bio03, Bio04, Bio08, Bio10) and two precipitation-related (i.e., Bio18, Bio19) were selected for linear model redundancy analysis (RDA). The optimal RDA model included three variables: Bio18, Bio04 and Bio03. The model was highly significant (*P* < 0.0001) and explained 20.17% (adjusted *R*^2^ = 0.2017) of the inherent genetic variation, indicating an important role of these environmental variables in shaping the distribution of AFLP genotypes. The first two RDA axes were significant and explained 19.82% and 8.10% of the variation, respectively. Variable Bio18 Precipitation of warmest quarter, significantly associated with RDA axis 1, contributed to the partitioning among northern, central and southern *H. italicum* populations. Both variables Bio03 Isothermality and Bio04 Temperature seasonality were associated with RDA axis 2 differentiating central *H. italicum* populations from the rest (Fig. 4).

## Discussion

Molecular analyzes of the genetic diversity and structure of *H. italicum* populations from the eastern Adriatic coast using AFLP markers revealed high intrapopulation diversity and low differentiation between populations as well as the population structure characterized by a pattern of isolation by distance. The dynamics of gene set in species is strongly influenced by life history traits such as life form, geographic range, reproductive system, seed dispersal mechanism and successional status^52–54^.

Recent studies have been conducted in the study area and have contributed significantly to the knowledge of species that are typical representatives of the Mediterranean climate, e.g., Dalmatian pyrethrum (*Tanacetum cinerariifolium* (Trevir.) Sch. Bip.)^55^ and sage (*Salvia officinalis* L.)^56^. Our results are comparable with studies on the above species, because the species have similar life traits. They are thermophilic, xerophytic, outcrossing perennials. Although these species generally have a wider range, their distribution pattern overlaps on the eastern Adriatic coast. The overall genetic diversity of *H. italicum* populations, indicated by the Shannon information index, showed intermediate values (0.355) compared to the populations of *S. officinalis* (0.387) and *T. cinerariifolium* (0.223). All three species are of great economic value, either as medicinal plants or, in the case of *T. cinerariifolium*, as a source of the potent natural insecticide pyrethrin. Overexploitation has been observed in all three species but may have most affected the genetic diversity of *T. cinerariifolium*, the species that has been extensively collected over long periods. In the case of *H. italicum*, collection of wild populations has increased more recently, in the last decade, and the impact on overall genetic diversity will become apparent in the future. Collection of immortelle from natural habitats is legally regulated in Croatia by the Nature Protection Act^57^ and the Ordinance on Collection of Native Wild Species^58^. Nevertheless, it is necessary to systematically monitor habitats, as harvesting regulations are often not respected. Results of the research showed that differentiation between populations of *H. italicum* was low, indicating extensive gene flow between populations^59^, and compared to *S. officinalis* and *T. cinerariifolium* it was the lowest. The AMOVA results showed greater intrapopulation genetic variation in *H. italicum*, which is typical for outcrossing plant species^54^. The greater genetic variation within populations compared to variation between populations was also observed in another outcrossing plant species from the family Asteraceae *Xanthium italicum* Moretti, naturally distributed on Corsica^60^. The similar partitioning of genetic diversity between and within populations was also found in the studies by Grdiša et al.^55^ and Jug-Dujaković et al.^56^.

The results of the model-based methods showed clustering of *H. italicum* populations into two distinct cluster: A, with populations from the north (Kvarner Bay) and B, with populations from the central and southern part of the study area (Dalmatia). These findings are congruent with the Fitch-Margoliash tree. The eastern Adriatic coast and the coastal Dinarides were heavily glaciated at various times during the Pleistocene^61–63^, which is well documented in the study of Quaternary sediments along the north of east Adriatic coast and the islands of Krk, Rab and Pag, as well as northern Dalmatia^63^. Much evidence suggests that glaciers were the barriers along the eastern Adriatic coast^64^ and may have been the reason for the separation of these two ancestral groups for a long time. We assume that the populations of *H. italicum* survived in the mini-refugia (microecologically favorable pockets) during various unfavorable climatic events and reconnected when climatic conditions became favorable again. This theory is confirmed by the existence of four refugia for the eastern Adriatic coast and the coastal Dinarides out of a total of 52 recognized putative refugia in the Mediterranean^65^. Many authors also confirmed the existence of several Pleistocene microrefugia for this study area^55,66–74^.

The separation of populations of *H. italicum* was found below the Kvarner Bay, near the Zrmanja river canyon (Obrovac), which is supported in a study by Marjanac and Marjanac^64^ that there is evidence of glaciation in the Obrovac area. Grdiša et al.^55^, also identified strong genetic differentiation of the north Adriatic genetic group for *T. cinerariifolium* with similar phylogeographic split at Kvarner Bay, near the Zrmanja canyon. In another study, Liber et al.^75^ found a distinct cluster at Kvarner Bay for *S. officinalis*, which also suggests the existence of refugia in the northern Adriatic. In the study by Rešetnik et al.^72^, a phylogeographic split is found between genetic clusters of *S. officinalis* located further south (at the border between northern and central Dalmatia). Glasnović et al.^73^, in their studies of *Edraianthus tenuifolius*, also indicated the possible presence of these two separate "refugia within refugia" during the LGM. The results can also be compared with studies of *Edraianthus tenuifolius*^68^, *Campanula pyramidalis*^76^ and *Cardamine maritime*^77^, which showed a similar phylogeographic split, but in the area of the Neretva Valley (Central Dalmatia). The rarity (DW value) in the northwestern *H. italicum* populations was higher than in the other parts of the study area, indicating long-term survival of the populations in the north^5^. The Shannon information index of *H. italicum* was also higher in the northern Adriatic populations. The high rarity of the northern Adriatic populations (Kvarner Bay) was also reported by Grdiša et al.^55^ for *T. cinerariifolium* and Liber et al.^75^ for *S. officinalis.* The lower DW values of *H. italicum* populations from the central and southern part of the study area might indicate later dispersal.

In 18 *H. italicum* populations, a total of 446 AFLP markers were used to identify loci thought to be under natural selection. A total of 21 loci that exhibited atypical values (*F*_*ST*_) (4.71%) were detected using the Mcheza program, of which 10 (2.24%) were under positive selection and 11 (2.47%) were under balancing selection, while nine (2.02%) loci under positive selection were identified using the BayeScan program. Based on the results of both programs, Mcheza and BayeScan, seven AFLP loci were under positive selection in *H. italicum.* Less than 5% of the large number of markers commonly examined by this method are identified as outliers^18^. Nosil et al.^25^ reported that 5–10% of loci are outliers when summarizing data from available studies. In the study of the species *Mikania micrantha* Kunth^78^, 14 outlier loci (2.9%) were identified using the Dfdist and BayeScan programs, while in the analysis of the species *Eruca sativa* Mill.^30^ nine loci were identified, but only three of them were confirmed by another method (1.6%). In mountain populations of *Sideritis scardica* Giseb.^79^, a lower number of loci were also identified under selection by BayeScan (3.10%) compared to Mcheza (5.31%). Similar results were obtained by other authors who emphasize the more conservative approach of the Bayesian method, that works more efficiently as it detects a larger number of outlier loci with a lower presence of false positives^80–82^. Therefore, it is recommended to use more methods that incorporate different approaches and use different algorithms to increase the probability that the detected loci are truly adaptive.

When adaptive loci are combined with climate variables, there is also the possibility of determining which climate factors are responsible for adaptive evolution^83^. The correlation between allele frequency variation and climatic variables were assessed by using the Spatial Analysis Method (Samβada), which does not depend on genetic models and operates at the individual level^84^. Using all three methods (Mcheza, BayeScan and Samβada), five (1.121%) loci under selection associated with bioclimatic traits were identified in *H. italicum* populations. Comparison with studies of other species confirms that only a small number of loci are potentially affected by natural selection. Müller et al.^33^ detected 11 (8.9%) outlier loci correlated to annual precipitation in the species *Geropogon hybridus* (L.) Sch. Bip. using the Mcheza, BayeScan and the spatial analysis method (SAM). Yang et al.^21^ used both frequency based (Dfdist and BayeScan) and correlation based (MLM) methods and showed that six outlier loci were strongly associated with at least one climate factor (temperature, precipitation, and radiation being the most important factors) in *Liriodendron chinense* (Hemsl.) Sarg. Authors Oberprieler et al.^34^ reported that using three methods (Mcheza, BayeScan, and Samβada) 732 AFLP loci screened revealed only 1.6% (full dataset) and 0.4% (reduced data set) of all loci were found to be under selection in *Diplotaxis harra* (Forssk.) Boiss. In the study of *Sideritis scardica* Giseb.^79^, seven outlier loci were identified by Mcheza, BayeScan, and Samβada and associated with bioclimatic variables with precipitation identified as a significant environmental factor driving adaptive genetic variation. Temperature and precipitation were the most important environmental factors triggering adaptation in *Rhododendron oldhamii*^85^, *Keteleeria davidiana* var. *formosana*^86^, *Salix* species^87^, and alpine species^11,88–91^. Another method, LFMM has been used to test gene-environment associations while estimating the effects of hidden factors that represent background residuals of population structure. The advantage of the LFMM method is that it has a low rate of false positives and negatives^92^, and it also offers the best compromise between detection capabilities and error rates when dealing with complex hierarchical neutral genetic structure^93^. The LFMM has recently been used in several studies. For example^94^ genetic divergence in *Pinus bungeana* Zucc. ex Endl. was investigated and six environmental variables were identified that were related to the ecological habitat of the species and were correlated with the highest number of environmentally associated loci. In the study of Li et al.^95^, annual mean temperature, annual precipitation and slope were considered to be the most important environmental factors associated with adaptive genetic divergence in *Cunninghamia konishii* Hayata. Our results obtained by combining *F*_*ST*_—outlier analysis (Mcheza and BayeScan) and genome-environment association analysis (Samβada, LFMM) showed that the most important environmental variables for adaptive genetic divergence in *H. italicum* populations on the eastern Adriatic coast were: Bio3 Isothermality, Bio08 Mean temperature of wettest quarter, Bio15 Precipitation seasonality, and Bio17 Precipitation of driest quarter. The observed natural populations of *H. italicum* inhabit sites under Mediterranean climate characterized by hot summers and cool winters with unevenly distributed precipitation prevailing in winter. Mediterranean plants cope with drought by developing different mechanisms that allows them to survive unfavorable or insufficient precipitation distribution^96^. In our study, the variable Bio15 Precipitation seasonality describes the differences between minimum and maximum precipitation values; therefore, our results suggest that *H. italicum* populations might be adapted to large fluctuations/amplitudes in the amount of precipitation. In Mediterranean climates, large rainfall amplitudes are common, and Mediterranean species strive to adapt and survive extremes. As Mediterranean region is highly vulnerable to climate change^96^ future projections for global climate change state that more uneven temporal distributions of precipitation are expected^97^. Climate models suggest that the hydrological cycle will intensify due to rising temperatures and increasing evaporation, leading to more storms and precipitation in particular areas, while drought will occur in areas far from storms^98^. According to Giorgi and Lionello^99^ and Matusik et al.^100^ the decrease of precipitation and increase of warming is expected in the future, together with increased inter-annual variability of both precipitation and temperature, which will cause greater frequency of extremely arid periods. Plant adaptation to seasonal water stress in Mediterranean climate is an important driver of genetic differentiation^96^.

The RDA approach used to study the contribution of bioclimatic variables to the genetic structure of natural populations of *H. italicum* also identified Bio03 Isothermality as important bioclimatic variable, together with Bio04 Temperature seasonality and Bio18 Precipitation of the warmest quarter as the main bioclimatic variables distinguishing northern, southern and central Adriatic populations. The variable Bio18 Precipitation of the warmest quarter contributed to the partitioning of the northern populations of *H. italicum*, as they are exposed to greater amounts of precipitation during the warmer months, leading to an adjustment of the populations in this direction. The two variables Bio03 Isothermality and Bio04 Temperature seasonality influenced the differentiation of the central *H. italicum* populations from the rest. Both variables are a measure of temperature heterogeneity, with Bio03 Isothermality quantifying how temperatures vary in relation to annual oscillations, while Bio04 temperature seasonality measures temperature changes throughout the year^101^. As mentioned above, the Mediterranean region is characterized by strong seasonality, both in temperature and precipitation, and the climate results in strong, opposing pressures on species^102^**.** Seasonality is an important component of climate that influences the availability of resources and thus the distribution of species in the environment at both temporal and spatial scales^103^. Central inland *H. italicum* populations cope with the wide range of daytime and nighttime temperatures, as well as cold winters and high summer temperatures, and are most likely adapted to temperature fluctuations, making them the most likely candidates for species persistence under ongoing climate change.

The development of numerous genome scanning and spatial statistical methods has facilitated analyzes and our knowledge of adaptability in non-model species, but there are some limitations to these methods. AFLPs are useful marker systems with high reproducibility and ability for discovering polymorphism without previous information of the genome^89^, but they are also poorly informative because they are dominant and biallelic, involving only reduced and anonymous part of the genome. The key weakness of genome scans is that they often detect false positives due to deviations from Hardy–Weinberg equilibrium and population structure model assumption^104^. Demographic events such as bottleneck, allele surfing during population expansion, secondary contact, and isolation by distance can mimic selection^12,105^, making it difficult to differ selection from demography^106^ and consequently to draw inferences about selection. Sampling strategy helps reduce occurrence of false positives by including abundant number of sampled individuals, more than 10 per site for allele frequency-based methods (e.g., *F*_*ST*_)^107,108^. Another way to reduce number of false positives and have more confidence in the outliers found is to use different approaches simultaneously and set strict thresholds, as was performed in this research. Four (0.89%) loci detected by all four methods (Mcheza, BayeScan, Samβada and, LFMM) are expected since only small number of them are affected by natural selection. These loci are possibly linked to genes under selection due to the the ‘hitchhiking effect’, but their location and function is unknown due to a lack of prior knowledge about the genome structure of the species under study. Therefore, this research is the first step in finding evidence for adaptive divergence of *H. italicum* and additional analyzes involving the discovery of the location and function of detected loci are needed. Adaptation to precipitation and temperature oscillations in *H. italicum* populations appears to be the most important trait to be further investigated using genotype–phenotype association studies.

The analyzes performed in this study and those proposed for future investigations could be the basis for future conservation strategies of *H. italicum* on eastern Adriatic coast. Extended sampling in the Mediterranean region should also be included in future analyses as the results obtained and conclusion derived in this study may not be applicable to the entire distribution range of the species. On the eastern Adriatic coast, in addition to longer-term problems such as degradation, habitat loss and climate change, increased demand for *H. italicum* has led to overharvesting, which could also lead to a long-term decline in genetic diversity. Evaluation of genetic diversity allows the identification of populations that have lower genetic diversity and are more vulnerable so conservation measures can be focused on them. Conservation of entire habitats through monitoring in the wild (in situ) can help legislators respond in a timely manner by adopting regulatory measures regarding the amounts of plant material allowed to be collected. Ex situ conservation should include seed banks with special attention to covering the most genetic diversity of the species^109^. Another possible solution is to promote the cultivation of *H. italicum* based on knowledge gained from molecular analysis and the adaptive potential of the species, which should be incorporated into breeding programs.

## Materials and methods

### Sampling and plant material

Leaf tissue from twenty-five individuals from 18 wild *H. italicum* populations was collected in July 2014 and 2015. In addition, the seed samples of each individual were collected and stored in the Collection of Medicinal and Aromatic Plants at the University of Zagreb, Faculty of Agriculture under the Accession numbers MAP02672-MAP02689 (data available at the Croatian plant Genetic Resources Database; https://cpgrd.hapih.hr/). The collection of plant and seed specimens was carried out within the National Programme for Conservation and Sustainable Use of Plant Genetic Resources for Food and Agriculture in the Republic of Croatia and therefore in accordance with relevant institutional, national, and international guidelines and legislation. Voucher specimens were identified by Tonka Ninčević and Marija Jug -Dujaković and are deposited in the ZAGR Virtual Herbarium, Zagreb, Croatia (available at: http://herbarium.agr.hr/; Herbarium IDs: 38977, 38981, 38983, 44230–44237, 59856–59862). Sampling sites are listed in Table 1. They were selected to represent the distribution range of *H. italicum* along the environmental gradient from northwest to southeast on the eastern Adriatic coast. Populations P01 to P06 were sampled in the northern part of the study area, populations P07–P10 in the central part and populations P11 to P18 in the southern part of the study area. The average distance between sampling sites was 145.83 km, with minimum and maximum distance of 7.93 km and 415.94 km, respectively. The climatic data on precipitation and temperature conditions of each sampling site were taken from the WorldClim database at a spatial resolution of approximately 1 km^2^^110^ (available at: www.worldclim.org). Generally, the average annual temperature increases from the northern to the southern parts of the study area, while the amount of precipitation decreases. Precipitation deficit becomes greater and lasts longer from the northern to southern part of the eastern Adriatic coast^111^.

### DNA extraction and AFLP fingerprinting

DNA samples were extracted from 23 to 25 individuals per each of the 18 populations. Total genomic DNA was isolated from 25 mg of silica gel dried leaf tissue using a DNeasy Plant Mini Kit (Qiagen®). DNA concentrations were measured using a P300 NanoPhotometer (Implen®).

The AFLP analysis was done as proposed in Vos et al.^112^ with minor modifications proposed in Carović-Stanko et al.^113^. Four combinations of selective primers were chosen for amplification: FAM-*EcoR*I-ACA + *Mse*I-CAC, NED-*Eco*RI-AGA + *Mse*I-CAC, VIC-*EcoR*I-ACG + *Mse*I-CGA and PET-*EcoR*I-AGC + *Mse*I-CGA. The amplified fragments were separated by capillary electrophoresis in an ABI3130xl Genetic Analyzer (Applied Biosystems®, Foster City, CA, USA).

### Data analysis

#### Within-population diversity

AFLP fragments were scored using the GeneMapper v. 4.0 software (Applied Biosystems) and fragments from 100 to 500 bp were analyzed. Error rates were estimated using the scanAFLP v. 1.2^114^. Genetic diversity within the populations was estimated by calculatating the percentage of polymorphic bands (%P), the number of private alleles (N_pr_) and the Shannon's information index (I)^115^. The Shannon’s information index was calculated as *I* =  − Σ (*p*_*i*_ log_2_ *p*_*i*_), where *p*_*i*_ is the phenotypic frequency^115^. The frequency down-weighted marker values (DW) were calculated according to Schönswetter and Tribsch^5^ using AFLPdat^116^ in R^117^.

#### Population differentiation and structure

Analysis of molecular variance (AMOVA^118^) was used to partition the total AFLP diversity between and within *H. italicum* populations. Analysis was performed using the program Arlequin v. 3.5.2.2^119^ and the significance of the *φ*_ST_ values was calculated based on 10,000 permutations. Pairwise population comparisons examined with AMOVA yielded a matrix of *φ*_ST_ values corresponding to the proportion of total variance shared between two populations that can be interpreted as the distance average between any two populations^120^.

Based on the frequency of amplified fragments of each AFLP marker in each analyzed population, allelic frequencies were calculated using the Bayesian method with a non-uniform prior distribution of allele frequencies according to Zhivotovsky^121^, implemented in AFLP-Surv v. 1.0^122^. We assumed that populations were in Hardy–Weinberg equilibrium (*F*_IS_ = 0) due to the outcrossing nature of *H. italicum*. The calculated allele frequencies were used to analyze genetic diversity within and between populations as described in Lynch and Milligan^123^. Total gene diversity (*H*_*T*_), average gene diversity within populations (*H*_*W*_), average gene diversity between populations that exceeds that observed within populations (*H*_*B*_), and Wright’s *F*_*ST*_ were used to describe the genetic structure of populations.

Standard Nei genetic distance (*D*_*NEI72*_) was calculated between all populations pairs of using AFLP-Surv v. 1.0^122^. An unrooted Fitch–Margoliash tree based on pairwise Nei’s standard genetic distances between populations was created using the FITCH program v. 3.6b (PHYLIP^124^). The bootstrap method was used to create 1,000 distance matrices using the AFLP-Surv, and the bootstrap values were calculated using the FITCH and CONSENSE programs within PHYLIP software package.

The genetic structure of *H. italicum* populations was assessed using two Bayesian model-based clustering methods as implemented in STRUCTURE v. 2.3.4^125^ and BAPS v. 6^126,127^. In STRUCTURE, the number of clusters (K) was set from 1 to 11 and 30 runs per K were performed on the Isabella computer cluster at the University of Zagreb, University Computing Centre (SRCE). An admixture model with correlated allele frequencies was applied with a burn-in period of 200,000 steps and 1,000,000 MCMC replicates after burn-in. The detection of the most likely number of clusters was performed as described by Evanno et al.^128^, and implemented in STRUCTURE HARVESTER v. 0.6.94^129^. Results from independent runs were clustered and averaged using Clumpak^130^ to obtain the Q-value matrix.

Another Bayesian model-based analysis was performed using BAPS^126,127^ to verify data obtained from STRUCTURE. Mixture analysis was performed both without the geographic coordinates as an informative prior (‘*Clustering of individuals*’) and with this prior (‘*Spatial clustering of individuals*’^131^. BAPS was run with a maximal number of clusters (*K*) set to 20 with each run replicated 10 times. The best *K* value was assessed using the log marginal likelihood values of the best partitions and the distribution of posterior probabilities for different *K* values. To detect admixture between clusters the results of the mixture analysis were used as input to the population admixture analysis^132^ with default settings.

Isolation by distance^133^ was performed by the Mantel's test based on 10,000 permutations using the program NTSys-pc v.2.10 s^134^ between the matrix of *F*_*ST*_ / (1−*F*_*ST*_) values and the matrix of natural logarithms of geographic distances (in km) between the analyzed populations.

#### Bioclimatic variation and adaptation

Values of 19 environmental variables (11 temperature-related and eight precipitation-related) representing the annual trends, seasonal variations, and extremes in temperature and precipitation, for each 18 *H. italicum* sampling site are shown in Table S3. A principal component analysis (PCA) was performed on 19 environmental variables and a biplot with two principal components (PC) showing sampled populations and environmental variables (as vectors) was constructed. To identify candidate loci under selection we applied: (i) a frequentist approach as described by Beaumont and Nichols^135^ and implemented in Mcheza^136^, (ii) a Bayesian approach as implemented in BayeScan v. 2.01^24^, and (iii) a spatial analysis method^27^ implemented in Samβada v. v0.5.1^84^, (iv) a latent factor mixed model (LFMM)^92^ implemented in R package lfmm^137^**,** and (v) a linear model redundancy analysis (RDA)^138^ implemented in R package vegan v. 2.5–7^139^**.** Analyzes were restricted to loci with dominant allele frequencies between 5 and 95% across the whole dataset to avoid a bias in global *F*_*ST*_ estimates^140^.

The methodology used in Mcheza is based on the assumption that loci under directional selection have significantly higher *F*_*ST*_ values than the majority of neutral loci in a sample. In contrast, loci under balancing selection are expected to exhibit significantly lower *F*_*ST*_ values. The neutral distribution of *F*_*ST*_ values was determined by 1,000,000 iterations using the 'Neutral mean *F*_*ST*_' and 'Force mean *F*_*ST*_ options. Outlier loci were selected with a confidence interval (CI) of 99% and a false discovery rate (FDR) of 0.1^135,136^. BayeScan evaluates individual loci within a hierarchical Bayesian model that decomposes genetic variation into population- and locus-specific effects. For each locus, two models are defined that include or exclude the effect of natural selection. The posterior probabilities of these two models are then estimated using a reversible-jump MCMC approach. We used 20 pilot runs with 5,000 iterations to fit the proposal distribution to acceptance rates between 0.25 and 0.45. Analyzes were performed with a burn-in of 50,000 iterations, a sample size of 10,000, and a thinning interval of 50, resulting in a total number of 550,000 iterations. The logarithm of Posterior Odds [log_10_(PO)] greater than 1.5 was considered as a 'very strong' evidence for selection^24,141^. The false discovery rate^142^ (FDR) was set to 0.01 to fit the corresponding log_10_(PO) significance threshold.

In Samβada, the spatial analysis method was used to calculate multiple univariate logistic regressions to test the probability of the presence of an allelic variant given the values of environmental variables of the sampling sites. Significance was assessed with both the log-likelihood G ratio and Wald test using Bonferroni correction (*P* < 0.01). A model was considered significant only if both tests rejected the corresponding null hypothesis^84^.

Gene-environment associations have also been identified using latent factor mixed models (LFMM^92,143^) implemented in the R package lfmm^137^. In LFMMs, associations between genetic variation and environmental variables are tested, while estimating the effects of hidden factors representing background residual levels of population structure. Thus, in LFMMs, environmental variables are introduced as fixed effects, while population structure is modeled by latent factors. The number of latent factors (K) was set to two based on the results of STRUCTURE and BAPS analyses. Regularized least squares were calculated using a ridge penalty (lfmm_ridge) and estimated the genomic inflation factor (GIF) was estimated based on median z-scores (lfmm_test). To account for multiple testing, p values were converted to q values using the R package qvalue^144^. Significant associations were selected based on a false discovery rate (FDR) of 5% (*q* < 0.05).

Linear model redundancy analysis (RDA), implemented in the R package vegan v. 2.5–7^139^, was used to analyse the effects of environmental variables on AFLP variation among populations. The highly correlated environmental variables (|r|> 0.70) were excluded before analysis. Hellinger-transformed allele frequencies^145^ were calculated in vegan based on the AFLP allele frequencies estimated using AFLP-Surv. The optimal model was determined using the ordiR2step function in vegan using a forward selection procedure with 10,000 permutations. To test the significance of the RDA model and constrained-axis, the vegan function anova.cca was run with 10,000 permutations.

## Supplementary Information

Supplementary Information.

## Data Availability

The datasets generated during and/or analyzed during the current study are available from the corresponding author on reasonable request.
