# Supplementary material for: Population structure and adaptive variation of Helichrysum italicum (Roth) G. Don along eastern Adriatic temperature and precipitation gradient
Source: Sci Rep. 2021 Dec 21;11:24333. doi: 10.1038/s41598-021-03548-6 (PMC8692458; doi:10.1038/s41598-021-03548-6)

Population structure and adaptive variation of *Helichrysum italicum* /Roth/ G. Don along eastern Adriatic temperature and precipitation gradient

Tonka Ninčević[1], Marija Jug-Dujaković[1], Martina Grdiša[2,3*], Zlatko Liber[3,4], Filip Varga[2,3], Dejan Pljevljakušić[5], Zlatko Šatović[2,3]

[1]Institute for Adriatic Crops and Karst Reclamation, Department for Plant Sciences, Put Duilova 11, 21000 Split, Croatia
[2]University of Zagreb, Faculty of Agriculture, Department of Seed Science and Technology, Svetošimunska c. 25, 10000 Zagreb, Croatia
[3]Centre of Excellence for Biodiversity and Molecular Plant Breeding (CoE CroP-BioDiv), Svetošimunska c. 25, 10000 Zagreb, Croatia
[4]University of Zagreb, Faculty of Science, Department of Biology, Marulićev trg 9a, 10000, Zagreb, Croatia
[5]Institute for Medicinal Plants Research "Dr Josif Pančić", Tadeuša Koščuška 1, 11000 Belgrade, Serbia

***Correspondence:**
Martina Grdiša
mgrdisa@agr.hr

Table S1. Interpopulation φ_{ST} values among 18 *Helichrysum italicum* populations from east Adriatic coast. Lower diagonal matrix: φ_{ST} values. Upper diagonal matrix: corresponding $P(\varphi_{ST})$ values.

No.	Population	P01	P02	P03	P04	P05	P06	P07	P08	P09	P10	P11	P12	P13	P14	P15	P16	P17	P18
P01	Krk		***	***	***	***	***	***	***	***	***	***	***	***	***	***	***	***	***
P02	Cres	0.018		***	***	***	***	***	***	***	***	***	***	***	***	***	***	***	***
P03	Lošinj	0.041	0.026		***	***	***	***	***	***	***	***	***	***	***	***	***	***	***
P04	Rab	0.036	0.041	0.047		***	***	***	***	***	***	***	***	***	***	***	***	***	***
P05	Pag 1	0.046	0.053	0.050	0.051		***	***	***	***	***	***	***	***	***	***	***	***	***
P06	Pag 2	0.079	0.074	0.060	0.073	0.060		***	***	***	***	***	***	***	***	***	***	***	***
P07	Obrovac	0.060	0.061	0.049	0.057	0.060	0.049		***	***	***	***	***	***	***	***	***	***	***
P08	Benkovac	0.078	0.092	0.103	0.101	0.100	0.104	0.085		***	***	***	***	***	***	***	***	***	***
P09	Kistanje	0.044	0.052	0.049	0.065	0.044	0.062	0.038	0.054		*	***	***	***	***	***	***	***	***
P10	Unešić	0.052	0.061	0.051	0.073	0.053	0.063	0.054	0.061	0.010		***	***	***	***	***	***	***	***
P11	Seget	0.077	0.074	0.086	0.092	0.091	0.094	0.092	0.023	0.041	0.044		***	***	***	***	***	***	***
P12	Brač	0.086	0.093	0.085	0.100	0.080	0.084	0.073	0.073	0.036	0.042	0.068		***	***	***	***	***	***
P13	Hvar	0.107	0.114	0.121	0.123	0.127	0.112	0.108	0.038	0.077	0.077	0.042	0.067		***	***	***	***	***
P14	Sinj	0.072	0.071	0.067	0.086	0.063	0.082	0.054	0.069	0.026	0.024	0.055	0.048	0.099		***	***	***	***
P15	Omiš	0.095	0.103	0.112	0.107	0.107	0.105	0.099	0.049	0.065	0.056	0.040	0.052	0.029	0.071		***	***	***
P16	Živogošće	0.083	0.089	0.091	0.096	0.083	0.090	0.076	0.070	0.039	0.043	0.057	0.035	0.057	0.053	0.044		***	***
P17	Slano	0.081	0.093	0.085	0.101	0.093	0.090	0.071	0.075	0.042	0.041	0.060	0.025	0.059	0.053	0.047	0.030		***
P18	Cavtat	0.070	0.083	0.084	0.075	0.078	0.073	0.056	0.085	0.062	0.073	0.080	0.066	0.076	0.081	0.063	0.056	0.054	

P-value significance level: $^{ns}P > 0.05$, *$0.01 < P < 0.05$, **$0.001 < P < 0.01$, ***$P < 0.001$.

Table S2. Correlations among 19 bioclimatic variables at 18 *Helichrysum italicum* sampling sites from the east Adriatic coast. Lower diagonal matrix: correlation coefficients (*r*). Upper diagonal matrix: corresponding *P*-values.

Bioclimatic variable	Bio01	Bio02	Bio03	Bio04	Bio05	Bio06	Bio07	Bio08	Bio09	Bio10	Bio11	Bio12	Bio13	Bio14	Bio15	Bio16	Bio17	Bio18	Bio19
Bio01		*	ns	ns	***	***	*	ns	***	***	***	ns	ns	ns	ns	ns	ns	ns	ns
Bio02	-0.469		***	ns	ns	***	***	ns	ns	ns	*	ns	ns	ns	ns	ns	ns	ns	ns
Bio03	-0.410	0.940		ns	ns	**	***	ns	ns	ns	ns	ns	ns	ns	ns	ns	ns	ns	ns
Bio04	-0.414	0.441	0.141		ns	*	***	ns	ns	ns	*	ns	ns	ns	ns	ns	ns	ns	*
Bio05	0.750	0.160	0.087	0.116		*	ns	ns	***	***	**	ns	ns	ns	ns	ns	ns	ns	ns
Bio06	0.942	-0.715	-0.618	-0.566	0.501		***	ns	***	***	***	ns	ns	ns	ns	ns	ns	ns	ns
Bio07	-0.521	0.933	0.768	0.731	0.166	-0.770		ns	ns	ns	**	ns	ns	ns	ns	ns	ns	ns	ns
Bio08	-0.149	0.038	0.046	-0.029	-0.209	-0.168	0.037		ns	ns	ns	ns	ns	**	**	ns	**	**	ns
Bio09	0.971	-0.412	-0.430	-0.191	0.828	0.878	-0.390	-0.155		***	***	ns	ns	ns	ns	ns	ns	ns	ns
Bio10	0.971	-0.412	-0.430	-0.191	0.828	0.878	-0.390	-0.155	1.000		***	ns	ns	ns	ns	ns	ns	ns	ns
Bio11	0.978	-0.543	-0.429	-0.589	0.624	0.972	-0.647	-0.137	0.905	0.905		ns	ns	ns	ns	ns	ns	ns	ns
Bio12	-0.098	0.012	0.183	-0.411	-0.276	-0.050	-0.146	0.197	-0.236	-0.236	-0.007		***	ns	ns	***	*	*	***
Bio13	-0.116	0.063	0.229	-0.407	-0.260	-0.067	-0.115	-0.009	-0.255	-0.255	-0.020	0.953		ns	ns	***	ns	ns	***
Bio14	-0.361	0.187	0.194	0.149	-0.297	-0.394	0.230	0.667	-0.363	-0.363	-0.374	0.435	0.176		***	ns	***	***	ns
Bio15	0.292	-0.095	-0.024	-0.316	0.194	0.323	-0.225	-0.666	0.227	0.227	0.343	0.149	0.412	-0.801		ns	***	***	**
Bio16	-0.104	0.079	0.247	-0.404	-0.239	-0.068	-0.098	0.053	-0.243	-0.243	-0.013	0.977	0.992	0.273	0.331		ns	ns	***
Bio17	-0.288	0.133	0.163	0.064	-0.272	-0.311	0.154	0.659	-0.307	-0.307	-0.290	0.563	0.309	0.984	-0.716	0.403		***	ns
Bio18	-0.288	0.133	0.163	0.064	-0.272	-0.311	0.154	0.659	-0.307	-0.307	-0.290	0.563	0.309	0.984	-0.716	0.403	1.000		ns
Bio19	0.141	-0.161	0.004	-0.505	-0.107	0.223	-0.333	-0.257	0.003	0.003	0.240	0.812	0.922	-0.160	0.680	0.889	-0.009	-0.009	

P-value significance level: $^{ns}P > 0.05$, $*0.01 < P < 0.05$, $**0.001 < P < 0.01$, $***P < 0.001$.

Table S3. Values of 19 bioclimatic variables of 18 *Helichrysum italicum* sampling sites from east Adriatic coast obtained from the WorldClim database[110]

No.	Bioclimatic variable	Sampling site (Population)																	
		P01	P02	P03	P04	P05	P06	P07	P08	P09	P10	P11	P12	P13	P14	P15	P16	P17	P18
Bio01	Annual Mean Temperature	145	131	140	138	139	134	129	122	129	131	129	157	146	136	149	150	142	132
Bio02	Mean Diurnal Range	81	75	79	89	92	100	100	92	94	84	79	77	75	83	82	83	83	82
Bio03	Isothermality	31	30	31	32	34	35	35	33	33	31	30	30	30	30	31	31	32	32
Bio04	Temperature Seasonality	6284	6194	6164	6355	6265	6291	6415	6494	6522	6476	6421	6193	6068	6603	6370	6320	6154	6011
Bio05	Max Temperature of Warmest Month	287	268	279	286	287	289	285	274	282	278	273	296	283	286	295	296	284	269
Bio06	Min Temperature of Coldest Month	28	18	25	16	17	8	2	-2	2	11	12	45	38	17	33	34	27	18
Bio07	Temperature Annual Range	259	250	254	270	270	281	283	276	280	267	261	251	245	269	262	262	257	251
Bio08	Mean Temperature of Wettest Quarter	112	100	109	105	106	101	94	87	95	98	97	96	87	101	84	84	78	102
Bio09	Mean Temperature of Driest Quarter	227	211	220	220	220	216	212	206	213	215	213	238	226	223	232	232	221	210
Bio10	Mean Temperature of Warmest Quarter	227	211	220	220	220	216	212	206	213	215	213	238	226	223	232	232	221	210
Bio11	Mean Temperature of Coldest Quarter	67	54	63	58	60	55	48	40	47	50	49	81	72	54	70	71	66	58
Bio12	Annual Precipitation	1289	1179	1050	1138	1089	1047	1020	1021	987	941	927	835	909	967	964	1092	1279	1301
Bio13	Precipitation of Wettest Month	158	147	133	147	141	139	137	137	133	127	124	111	121	129	130	150	178	182
Bio14	Precipitation of Driest Month	72	65	56	59	55	51	51	53	51	47	46	35	37	45	39	39	40	40
Bio15	Precipitation Seasonality	27	26	26	29	30	31	30	30	31	31	31	33	34	31	34	37	40	39
Bio16	Precipitation of Wettest Quarter	456	410	367	413	397	388	379	379	367	348	341	304	336	350	355	413	487	493
Bio17	Precipitation of Driest Quarter	241	218	190	200	187	175	173	175	169	160	156	128	134	163	145	148	154	154
Bio18	Precipitation of Warmest Quarter	241	218	190	200	187	175	173	175	169	160	156	128	134	163	145	148	154	154
Bio19	Precipitation of Coldest Quarter	342	314	283	310	300	291	286	289	281	276	275	263	296	294	310	367	433	429

Figure S1. The choice of the most likely number of clusters (*K*) inferred from multilocus AFLP data of 18 *Helichrysum italicum* populations from east Adriatic coast using Structure[115]: *ln P*(X|K) values for each of the 30 independent runs for each *K* and Δ*K* values for each *K*[128]

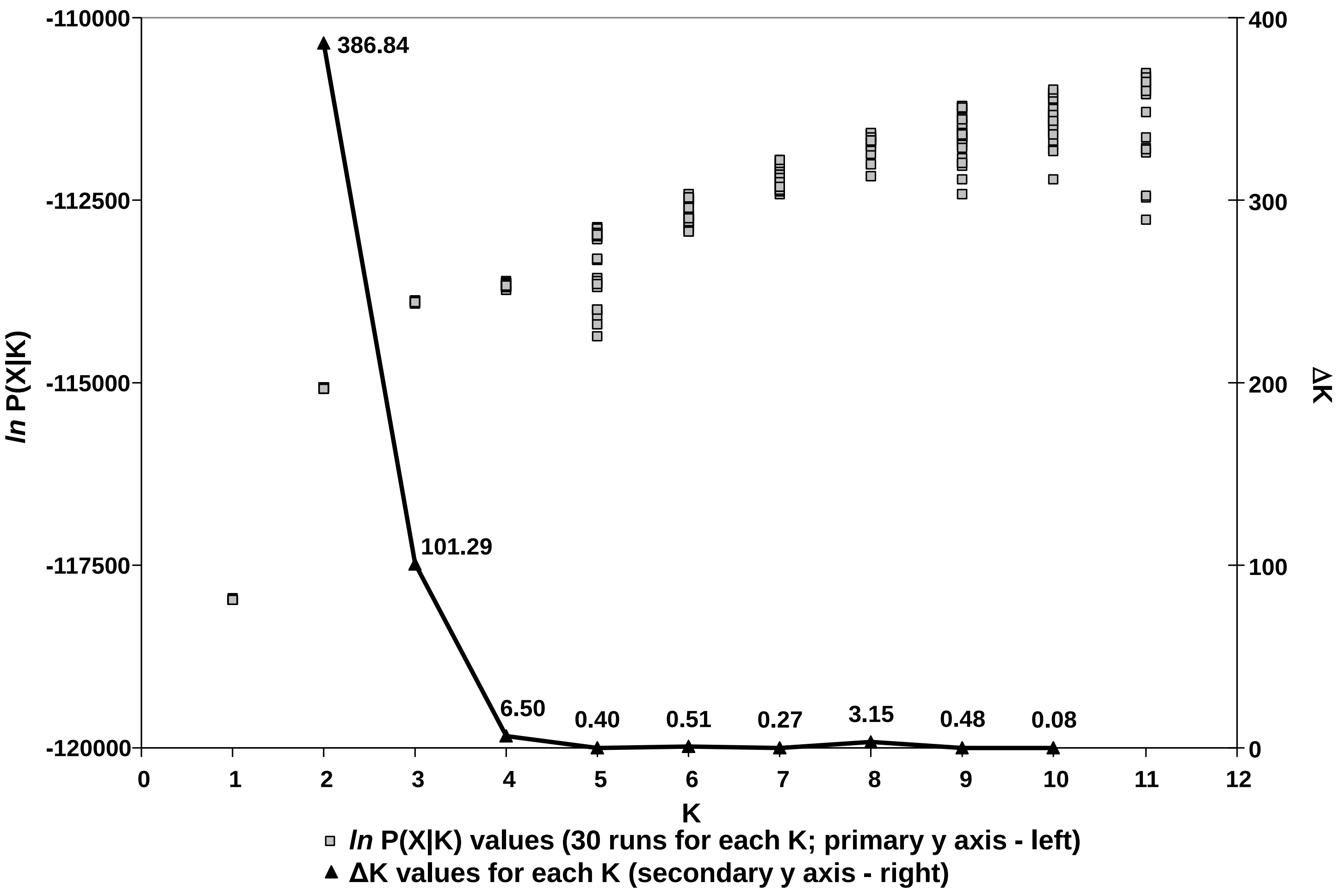

Figure S2. Population structure of 18 *Helichrysum italicum* populations from east Adriatic coast as inferred using BAPS[127] (A) without spatially informative prior, and (B) with spatially informative prior. Each individual is represented by a column, and the color corresponds to the percentage (i.e. *Q*-values) of the individual belonging to a particular ancestral population.

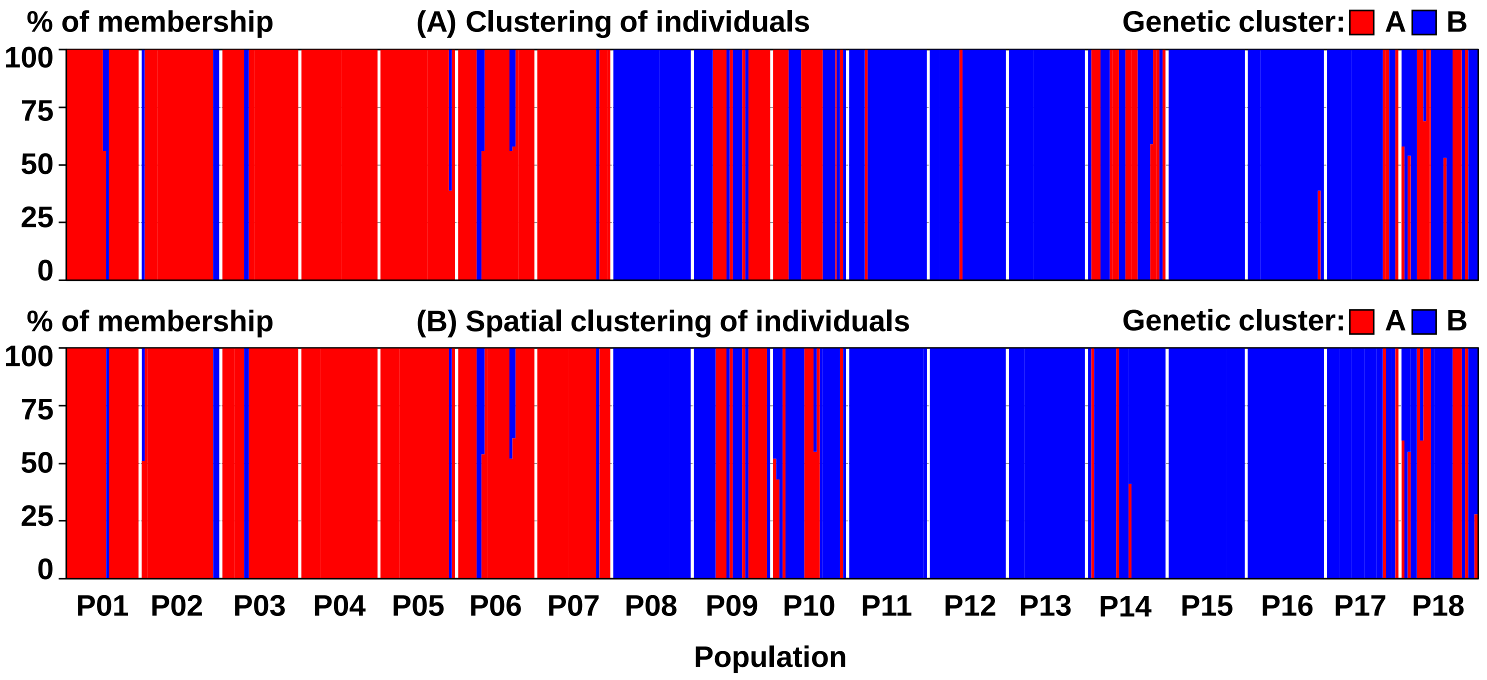

Figure S3. Isolation-by-distance analysis of 18 *Helichrysum italicum* populations from east Adriatic coast: regression of genetic distance [F_{ST} / (1-F_{ST})] on geographical distance [*ln*(km)] among sampling sites.

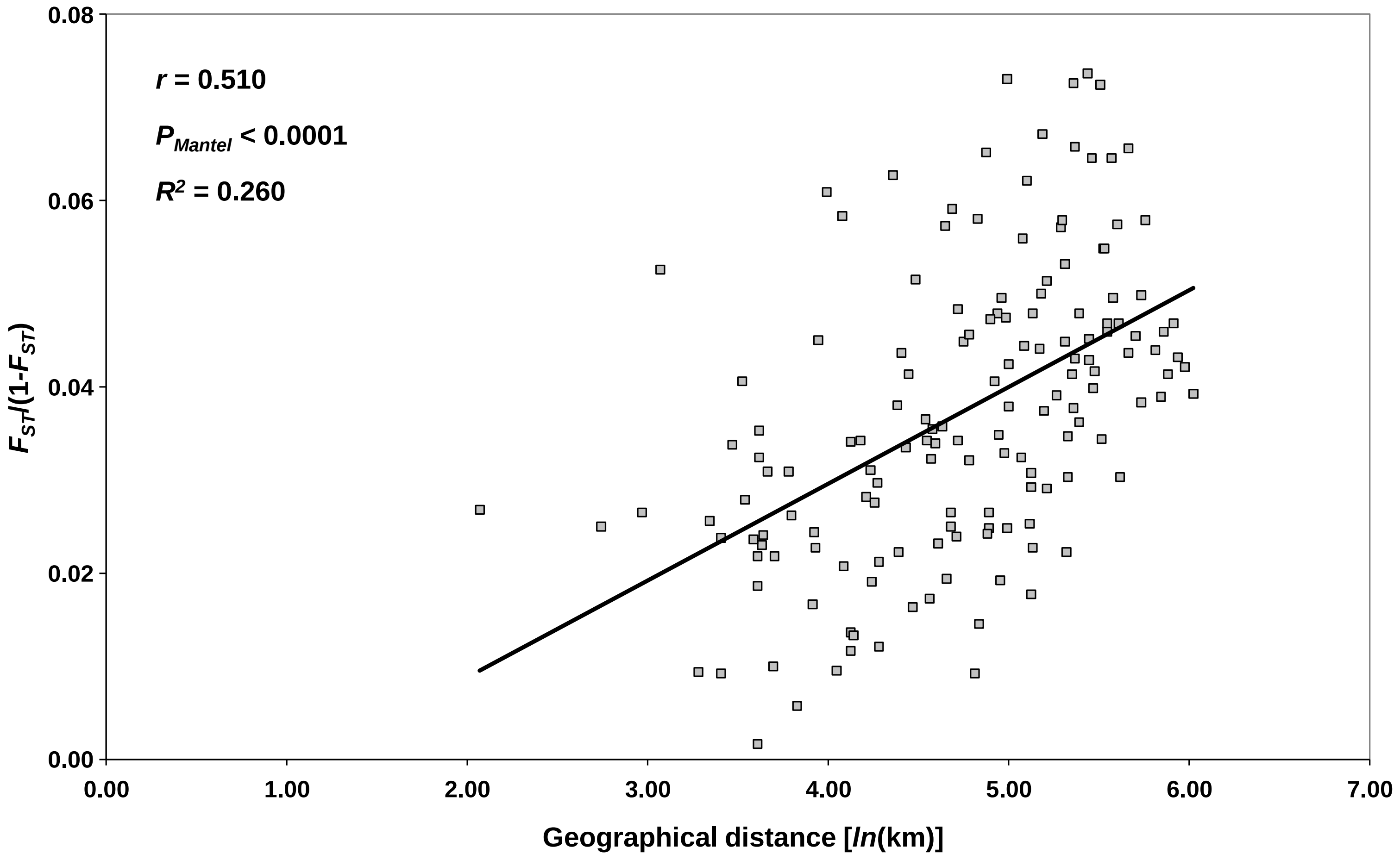

Supplement: Supplementary file 1 — Supplementary Information. [file 41598_2021_3548_MOESM1_ESM.pdf]
